# Beyond Conventional Frenectomy: A Case Series

**DOI:** 10.7759/cureus.109233

**Published:** 2026-05-19

**Authors:** Manisha Kumari, Vineeta Gupta, Shirish K Kujur, Jaishree Borkar, Pranav Sanjay Aher, Tanisha Shrivastava, Arunkumar P Dodamani

**Affiliations:** 1 Department of Periodontics, Government Dental College, Raipur, Raipur, IND

**Keywords:** aberrant frenum, frenectomy, midline diastema, periodontal plastic surgery, v-y plasty, z-plasty

## Abstract

Persistent midline diastema in adolescents is often associated with an aberrant high labial frenum requiring surgical correction. Advanced frenectomy techniques such as Z-plasty and V-Y plasty may provide favorable esthetic and functional outcomes in selected cases; however, larger studies with longer follow-up are required for definitive comparison with the conventional approach. These techniques enable improved tissue repositioning, reduced tension, minimal scar formation, and enhanced vestibular depth, thereby improving esthetic outcomes. This case series evaluates and compares conventional frenectomy with established advanced techniques, Z-plasty and V-Y plasty, in the management of abnormal labial frenum attachments. All techniques demonstrated satisfactory short-term healing and functional outcomes during the one-month follow-up period; however, the conventional method showed comparatively greater scar formation, whereas advanced techniques exhibited clinically favorable esthetic outcomes during short-term follow-up, improved tissue adaptation, and better functional results. Within the limitations of this case series, advanced frenectomy techniques may provide improved outcomes, and the choice of technique should be individualized based on clinical presentation and esthetic requirements.

## Introduction

Increasing esthetic awareness and patient expectations have contributed to a greater demand for periodontal plastic surgical procedures aimed at improving both function and esthetics. The frenum is a fold of mucous membrane containing muscle and connective tissue fibers that attach the lips and cheeks to the gingiva, periosteum, and alveolar mucosa. Clinical attention to the labial frenum has become essential, as an aberrant frenum is considered an important etiological factor contributing to the persistence of midline diastema [1].

Placek et al. proposed a morphological classification of the maxillary labial frenum based on its site of attachment, categorizing it into mucosal, gingival, papillary, and papilla-penetrating types [2]. Among these, papillary and papilla-penetrating types are frequently associated with pathological conditions such as midline diastema, gingival recession, and compromised plaque control. An abnormal or high frenum attachment may exert a pull on the gingival margin, leading to mucogingival problems and difficulty in maintaining oral hygiene, thereby adversely affecting periodontal health [1,3].

Management of an aberrant frenum can be achieved through surgical procedures such as frenotomy and frenectomy. Frenotomy involves incision and relocation of the frenal attachment, whereas frenectomy entails complete excision of the frenum along with its attachment to the underlying bone. Various surgical techniques have been described, including conventional scalpel methods, electrosurgery, laser-assisted approaches, V-Y plasty, and Z-plasty.

## Case presentation

Case presentation 1

**Figure 1:**
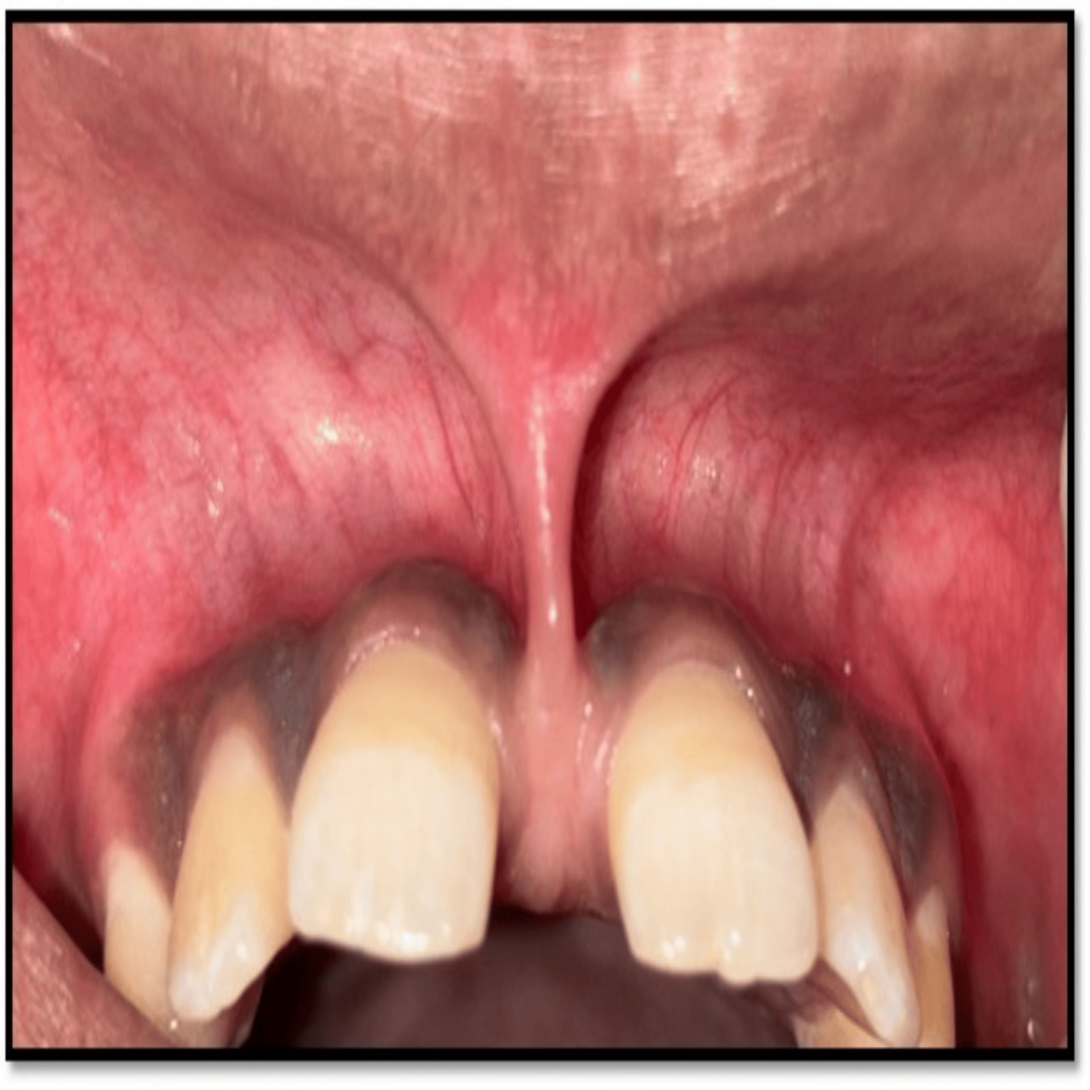
Case 1: Preoperative intraoral view showing maxillary midline diastema associated with a high papillary-type labial frenum attachment extending into the interdental papilla between the maxillary central incisors.

A 15-year-old male patient was referred from the Department of Orthodontics to the Department of Periodontics with a chief concern of spacing between the upper front teeth. Clinical examination revealed a maxillary midline diastema associated with a high papillary-type labial frenum extending into the interdental papilla between the maxillary central incisors (Figure 1). The blanch test was positive, confirming the aberrant frenal attachment.

The patient was planned for frenectomy as part of an interdisciplinary approach prior to orthodontic closure of the diastema. A detailed medical history was obtained, which was non-contributory. Written informed consent was obtained from the patient and guardian, and routine hematological investigations were within normal limits. Following phase I therapy with oral prophylaxis, the surgical procedure was performed under local anesthesia with strict aseptic precautions.

**Figure 2:**
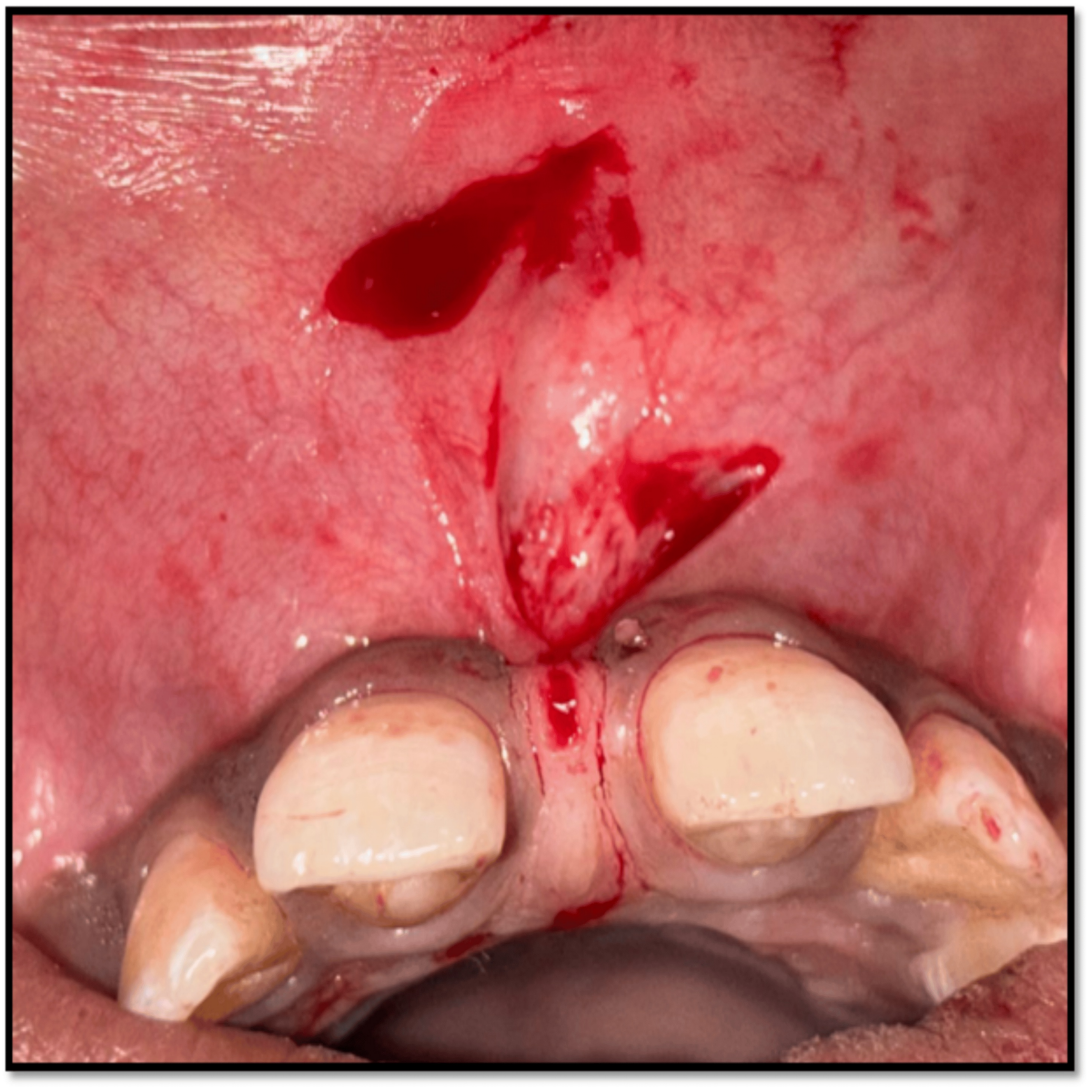
Case 1: Intraoperative view showing Z-plasty incision design with a central vertical incision along the frenal attachment and two oblique incisions placed at approximately 60° angles, forming triangular flaps for transposition.

**Figure 3:**
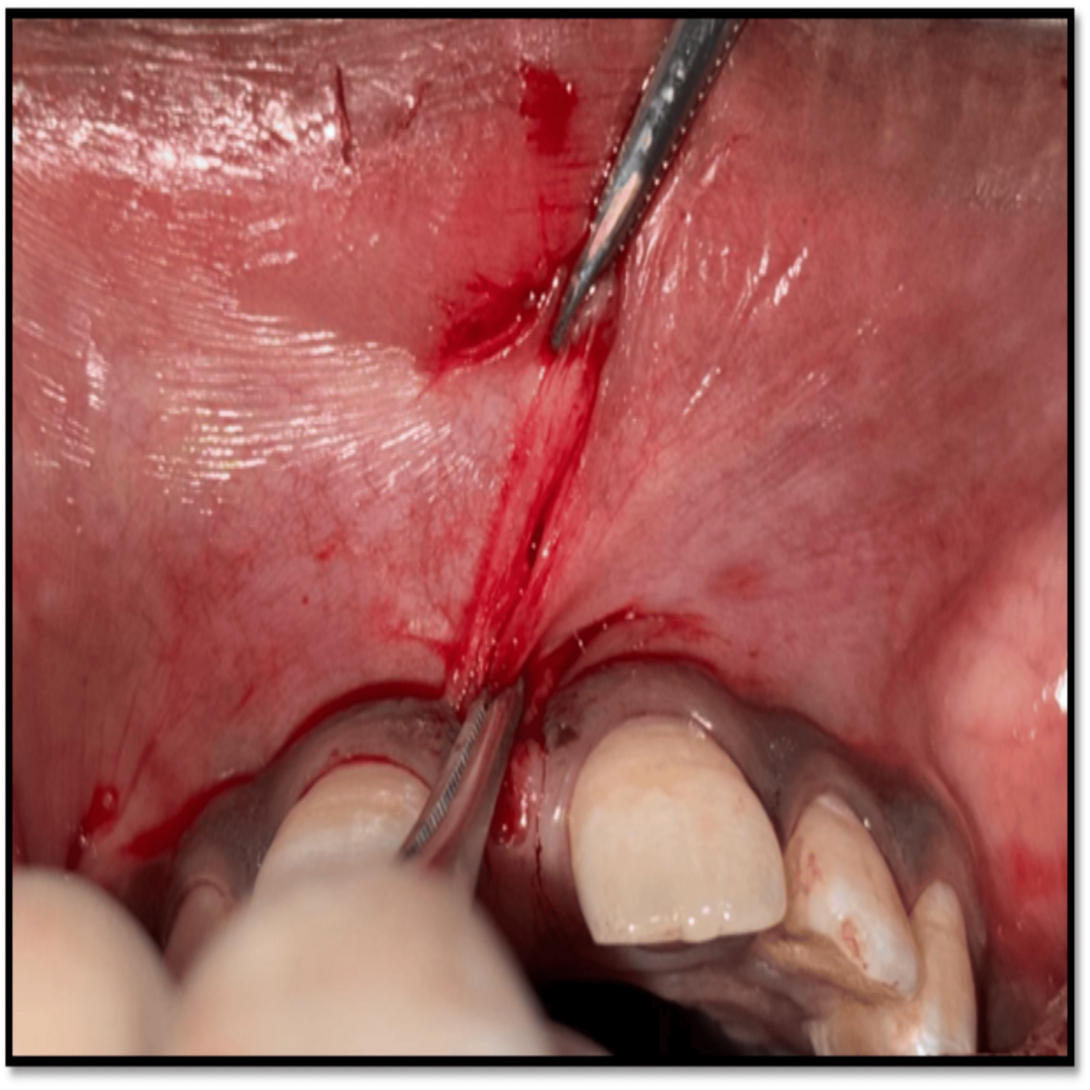
Case 1: Following hemostasis, the triangular flaps were transposed to the opposite sides of their respective apices.

A Z-plasty incision design was utilized, consisting of a central vertical incision along the frenal attachment and two oblique incisions placed at approximately 60° angles to create triangular flaps of equal size (Figure 2). These flaps were carefully elevated, transposed, and sutured in their new positions to achieve adequate vestibular depth and release of tension. Adequate undermining of the surrounding tissues was performed to minimize distortion of the underlying structures and allow tension-free flap placement. Following the achievement of hemostasis, the triangular flaps were transposed to the opposite sides of their respective apices, as illustrated in Figure 3. Hemostasis was achieved, and the surgical site was closed using non-resorbable sutures.

Postoperative instructions were provided, and the patient was prescribed analgesics along with oral hygiene maintenance advice. Healing was uneventful, with satisfactory functional and esthetic outcomes observed during follow-up. The patient has been planned for subsequent orthodontic treatment to achieve closure of the midline diastema.

**Figure 4:**
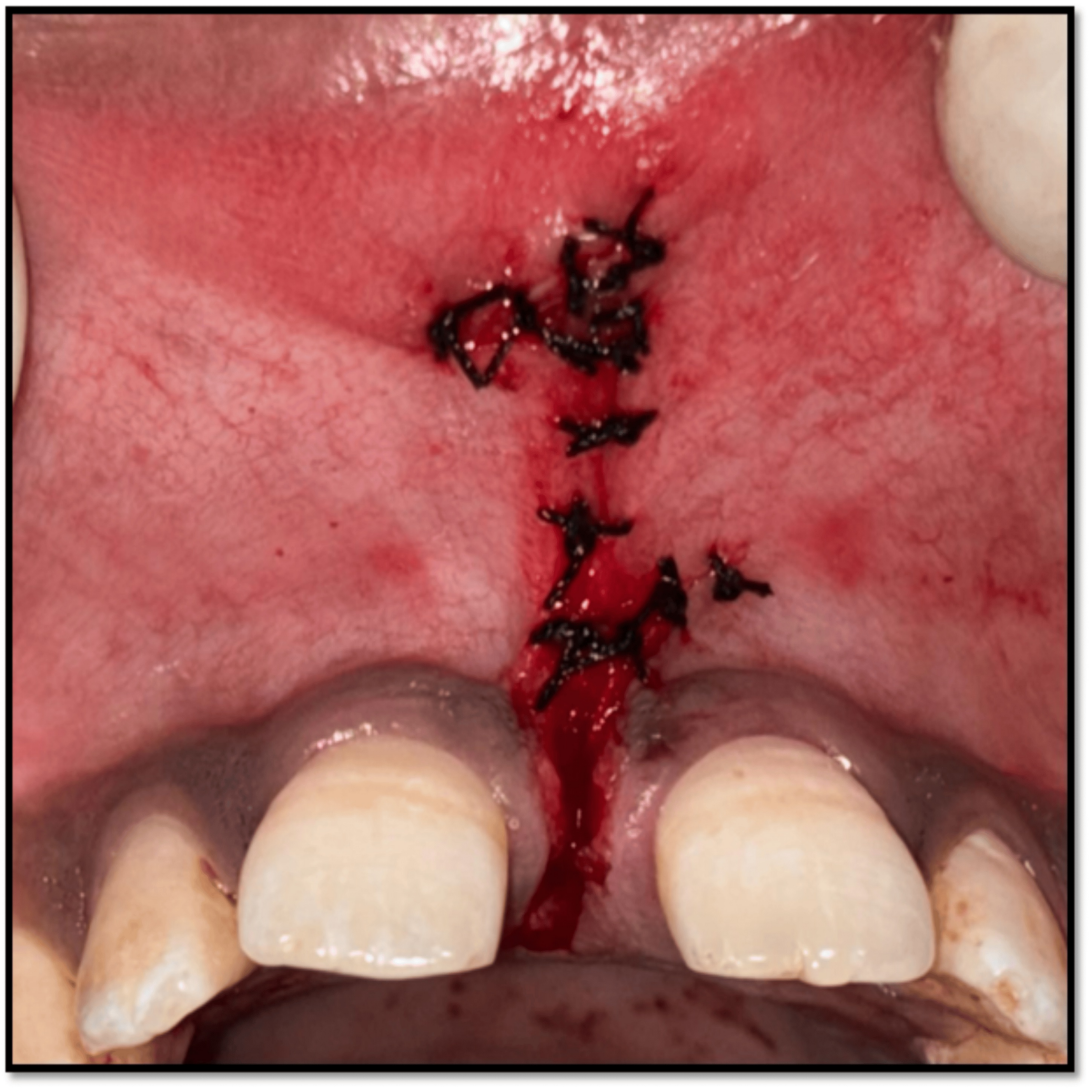
Case 1: Intraoperative view showing repositioned triangular flaps secured with sutures in their transposed positions.

**Figure 5:**
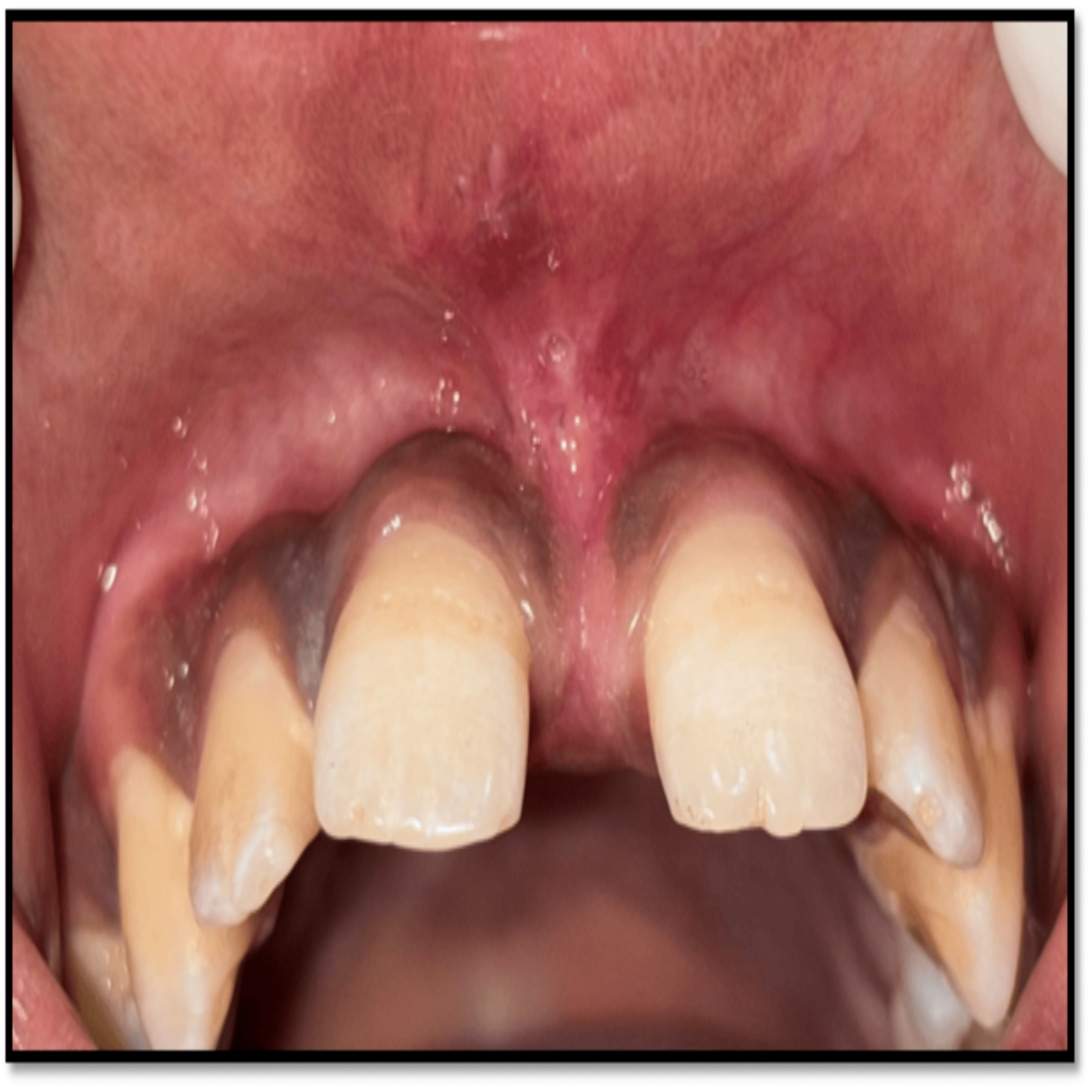
Case 1: Postoperative view at one week following suture removal, demonstrating satisfactory healing without complications.

The flaps were then repositioned and secured in their new orientation by suturing them to the recipient sites on the opposite sides of their bases, as shown in Figure 4. The patient was recalled after seven days for suture removal, which revealed satisfactory healing without any discomfort (Figure 5). At the one-month follow-up, the Z-plasty frenectomy site demonstrated complete healing with no clinically visible scar formation and favorable outcomes from both orthodontic and periodontal perspectives. Z-plasty, a well-established plastic surgical technique, involves the creation and transposition of triangular flaps to redistribute tension, improve tissue length, and minimize scar formation, thereby providing improved functional and esthetic outcomes [4,5].

Case presentation 2

**Figure 6:**
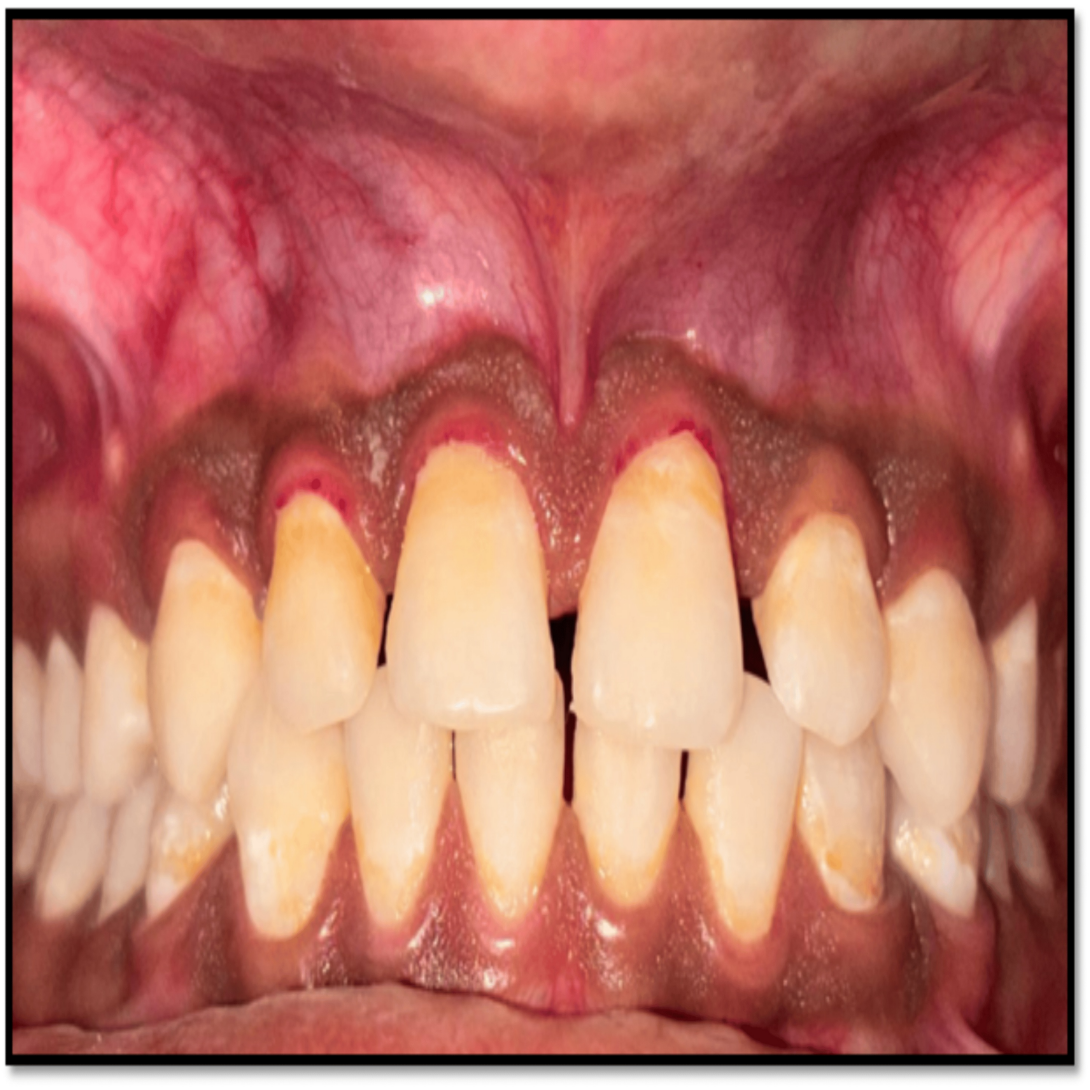
Case 2: Preoperative intraoral view showing aberrant labial frenum indicated for V-Y plasty technique.

An 18-year-old male patient presented with spacing between the upper anterior teeth. Clinical examination revealed a maxillary midline diastema associated with an aberrant labial frenum attachment (Figure 6). The blanch test was positive, confirming pathological frenal involvement.

**Figure 7:**
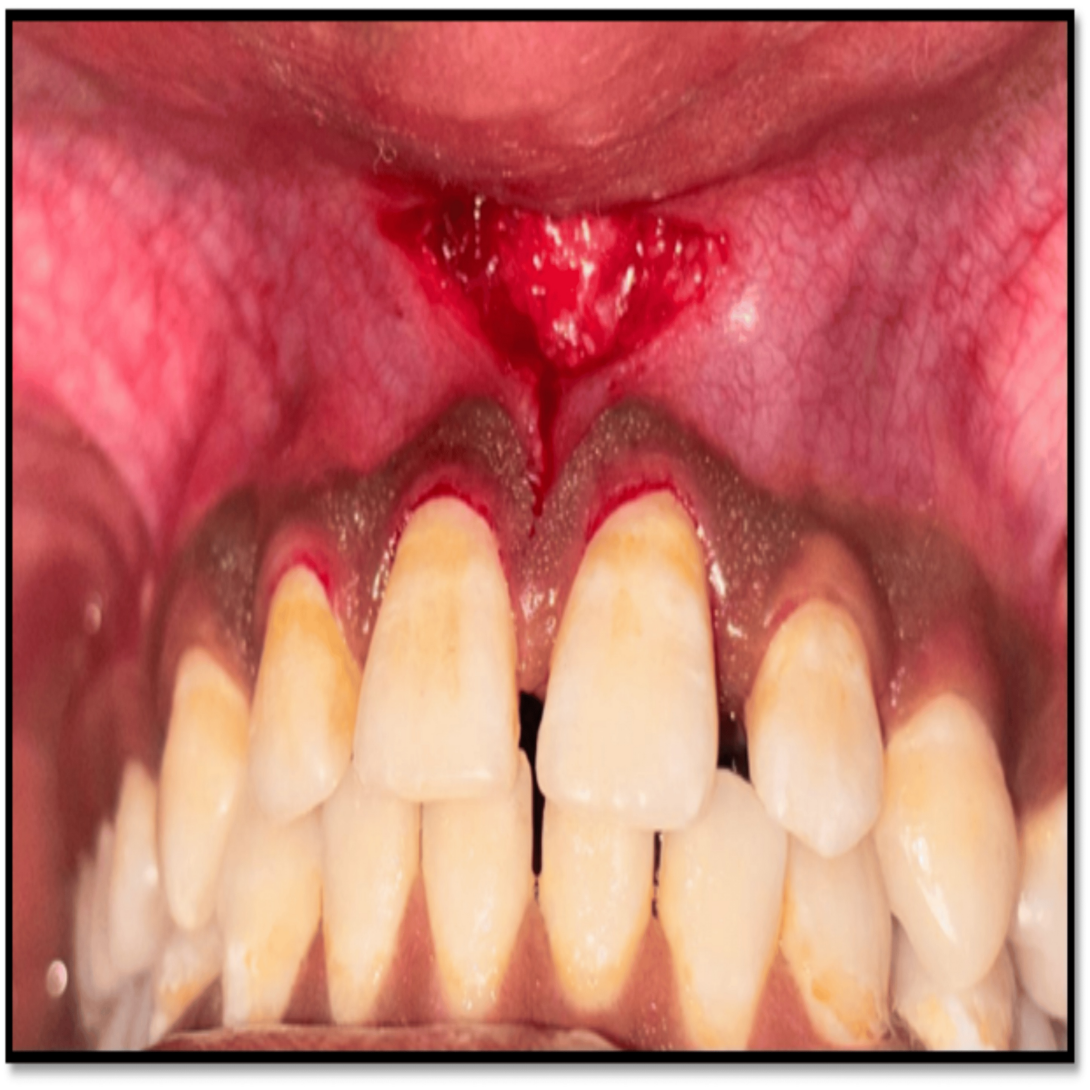
Case 2: Intraoperative view showing incision of the frenum in a V-shaped pattern for V-Y plasty.

**Figure 8:**
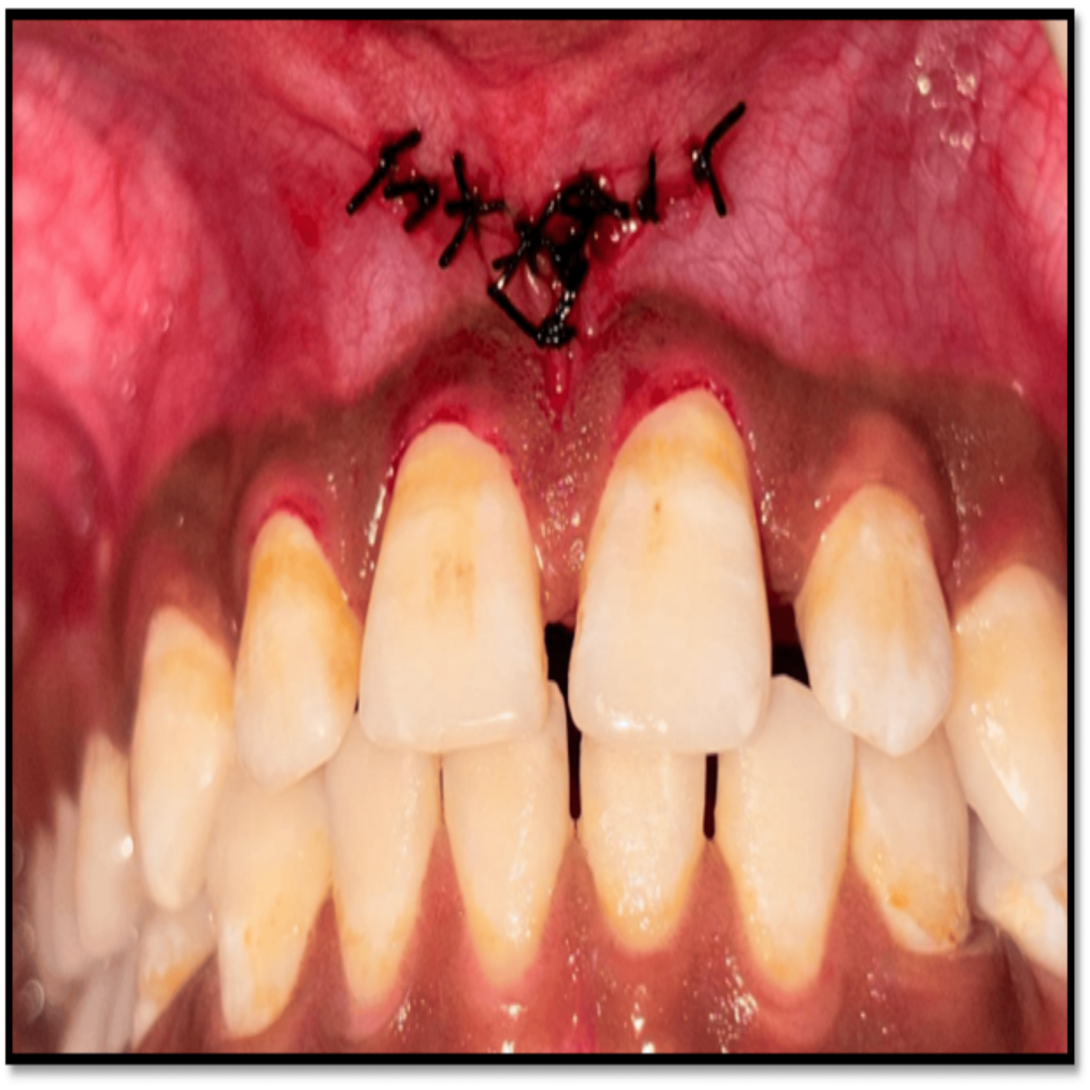
Case 2: Intraoperative view showing suturing of the surgical site in a Y configuration following V-Y plasty, achieving apical repositioning of the frenum.

Frenectomy was planned as part of an interdisciplinary approach prior to orthodontic correction. Following initial therapy, a V-Y plasty technique was performed. A V-shaped incision was placed around the frenal attachment extending into the vestibule (Figure 7). The fibrous tissue was dissected and released, and the surrounding mucosa was undermined to facilitate tension-free repositioning. The incision was then approximated and sutured in a Y configuration (Figure 8), resulting in apical repositioning of the frenum and increased vestibular depth.

Healing was uneventful, with satisfactory reduction in frenal pull and improvement in vestibular depth observed during follow-up. At one week, healing was satisfactory following suture removal, and at one month, minimal scar formation with favorable functional and esthetic outcomes was noted. The patient was subsequently referred for orthodontic management of the midline diastema.

Case presentation 3

**Figure 9:**
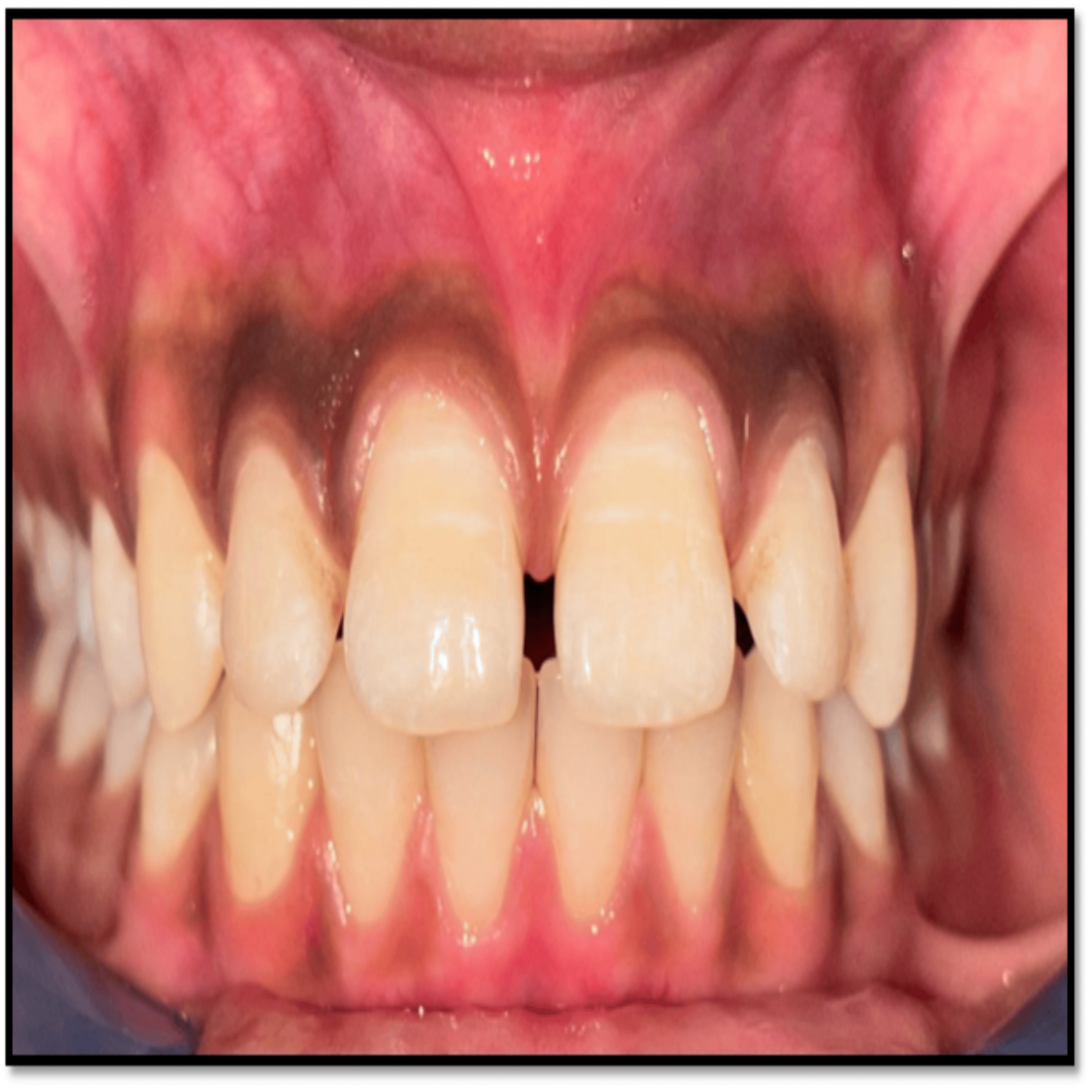
Case 3: Preoperative intraoral view showing aberrant labial frenum indicated for conventional frenectomy.

A 14-year-old male patient presented with spacing between the upper anterior teeth. Clinical examination revealed a maxillary midline diastema associated with an aberrant labial frenum attachment (Figure 9). The blanch test was positive, confirming pathological frenal involvement.

**Figure 10:**
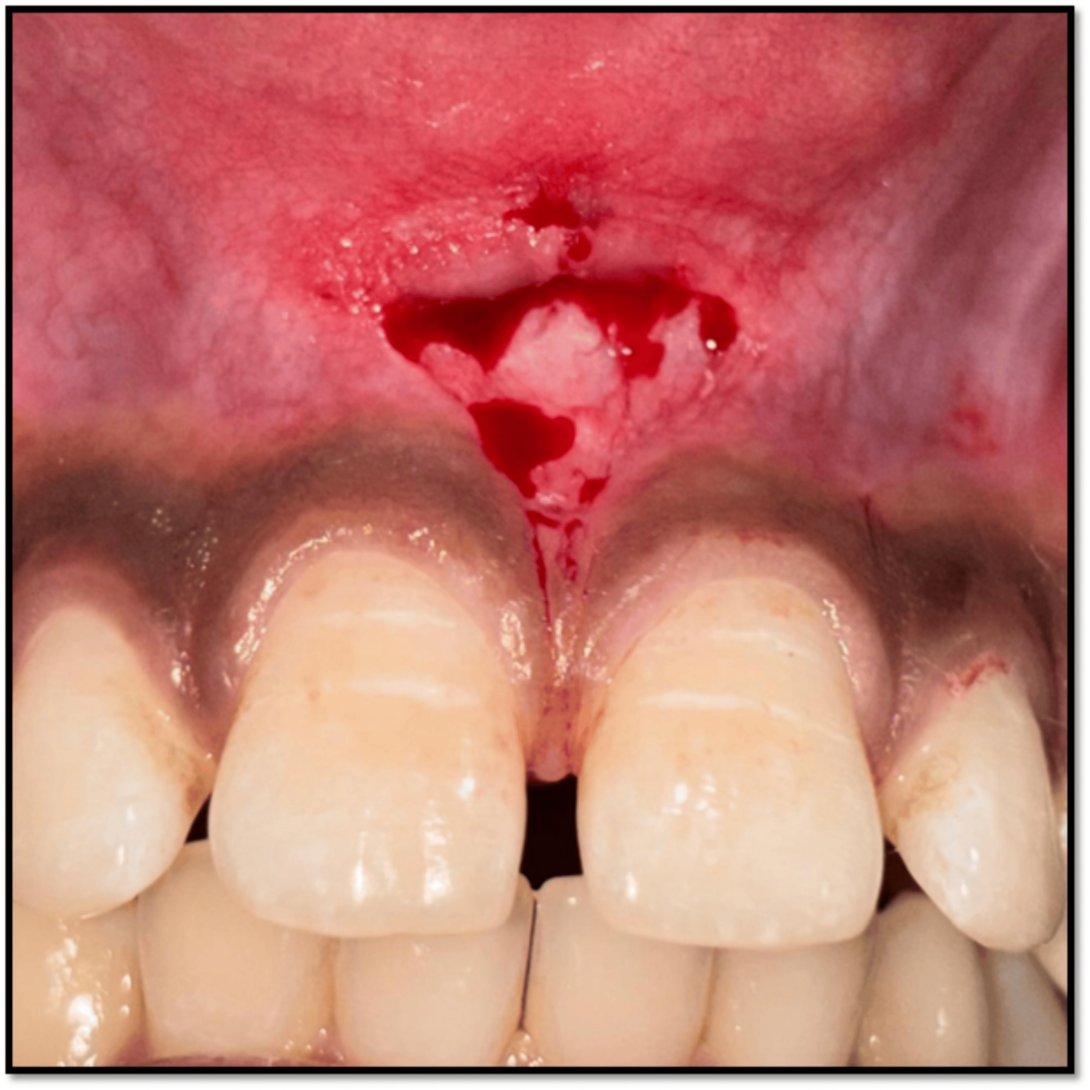
Case 3: Intraoperative view showing diamond-shaped incision performed using a hemostat for conventional frenectomy.

**Figure 11:**
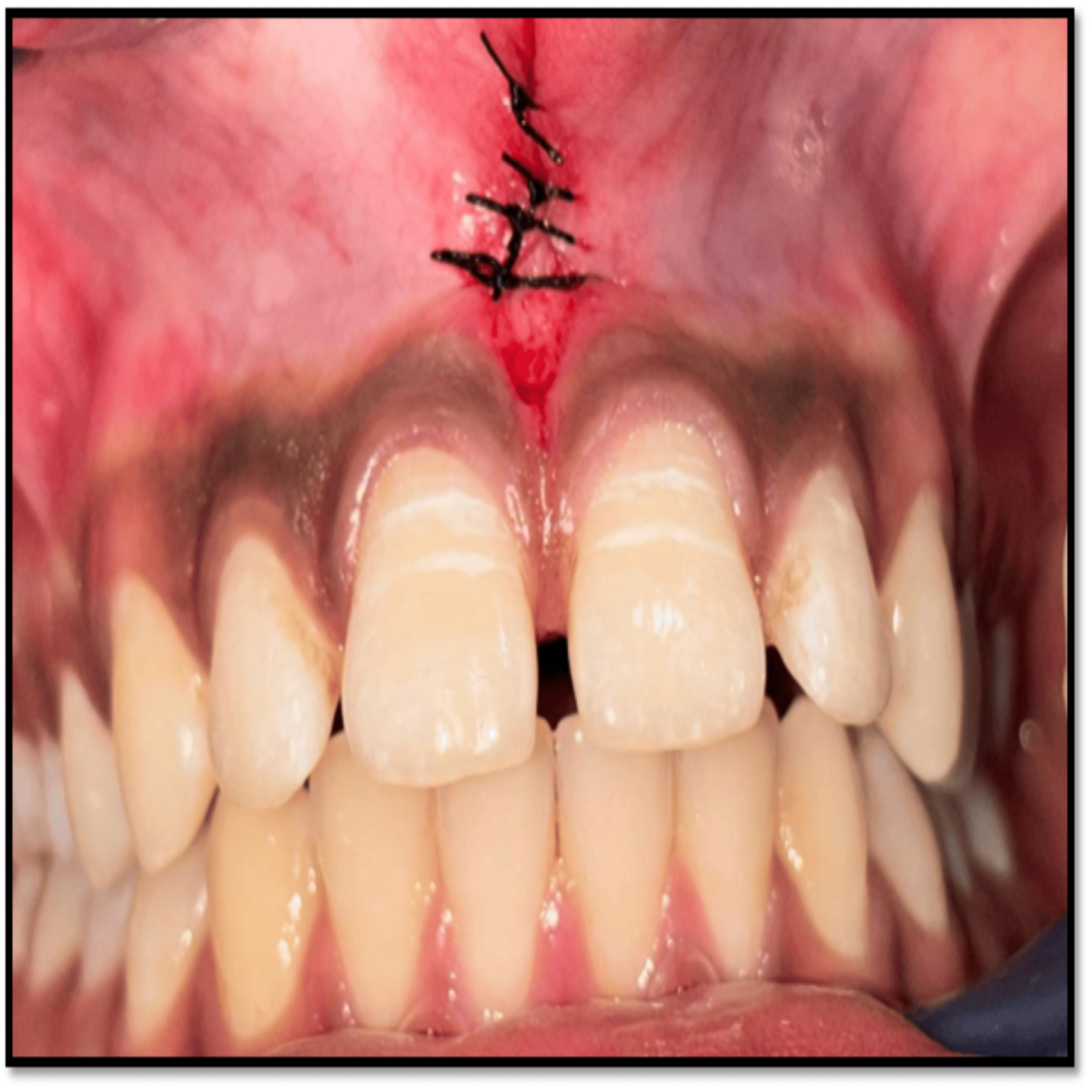
Case 3: Intraoperative view showing suturing of the surgical site following conventional frenectomy.

Frenectomy was planned as part of an interdisciplinary approach prior to orthodontic correction. Following initial therapy, a conventional frenectomy technique was performed. The frenum was engaged using a hemostat, and a diamond-shaped incision was placed to excise the fibrous attachment (Figure 10). The underlying fibrous tissue was completely removed to ensure adequate release of the frenal pull. Blunt dissection was carried out to separate muscle fibers and minimize the risk of reattachment. Hemostasis was achieved, and the surgical site was approximated and sutured using non-resorbable sutures (Figure 11).

**Figure 12:**
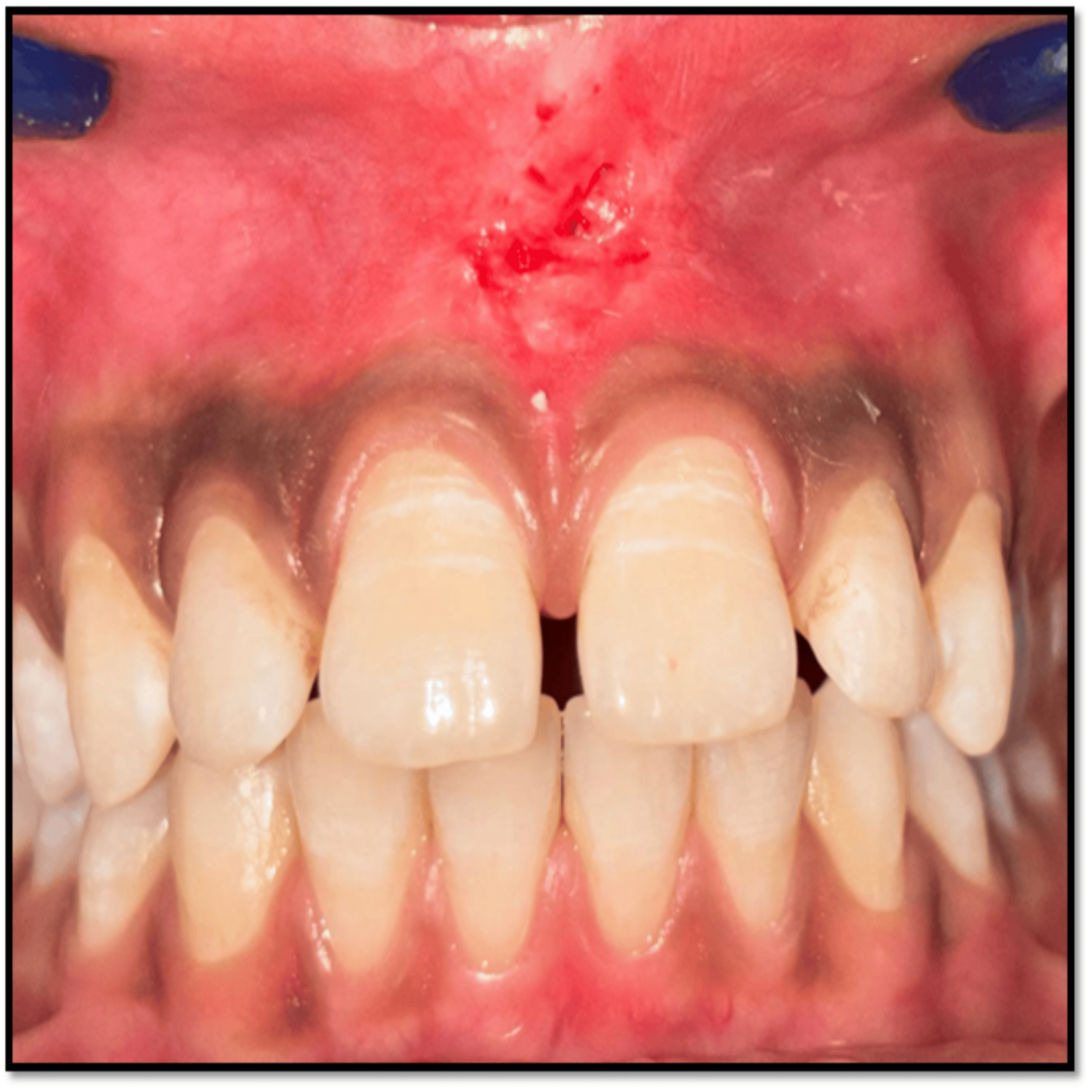
Case 3: Postoperative view at one week following conventional frenectomy, demonstrating satisfactory healing of the surgical site following suture removal.

Healing was uneventful, with satisfactory reduction in frenal pull observed during follow-up. At one-week follow-up, satisfactory healing was noted following suture removal (Figure 12). At one month, the surgical site demonstrated acceptable healing, although mild scar formation was observed. The patient was subsequently referred for orthodontic management of the midline diastema.

## Discussion

Various techniques have been described for the management of aberrant labial frenum, including conventional scalpel frenectomy, Z-plasty, V-Y plasty, electrosurgery, and laser-assisted procedures [5,6]. Among these, periodontal plastic surgical approaches such as Z-plasty and V-Y plasty are considered advanced techniques due to their ability to provide superior functional and esthetic outcomes compared to conventional methods.

The conventional frenectomy technique, although simple and widely practiced, involves complete excision of the frenum and healing by secondary intention, which may result in scar formation and reduced vestibular depth. In the present case series, the conventional approach demonstrated satisfactory elimination of frenal pull; however, mild scar formation was observed, consistent with previous reports [7,8]. Such scarring may compromise esthetics, particularly in the anterior maxillary region.

Z-plasty, a well-established plastic surgical technique first described by Denonvilliers in 1856, has been widely utilized in reconstructive surgery for scar revision and tissue lengthening [9,10]. It involves the creation and transposition of triangular flaps, typically designed at 60° angles, to redistribute tension, reorient scars, and increase tissue length. In the context of frenectomy, Z-plasty facilitates healing by primary intention, improves vestibular depth, and minimizes scar formation by aligning the incision along natural tension lines. In the present case series, Z-plasty demonstrated excellent healing with no visible scar formation and clinically favorable esthetic outcomes, making it particularly advantageous in cases with high esthetic demand.

V-Y plasty is another advanced technique that focuses on apical repositioning of the frenum through a V-shaped incision that is sutured in a Y configuration. This technique effectively lengthens the mucosa, increases vestibular depth, and reduces muscle pull, thereby minimizing the risk of relapse and scar contracture [11,12]. In the present series, V-Y plasty resulted in satisfactory healing, improved vestibular depth, and minimal scar formation, supporting its effectiveness as a functional and esthetic alternative to conventional frenectomy.

The findings of this case series highlight that while all techniques are effective in eliminating aberrant frenal attachments, advanced plastic surgical techniques such as Z-plasty and V-Y plasty provide superior outcomes in terms of esthetics, tissue adaptation, and vestibular depth. These techniques promote healing by primary intention and reduce postoperative scar formation, which is particularly important in the anterior esthetic zone.

However, it is important to note that these advanced techniques are more technique-sensitive and require careful case selection, precise surgical execution, and adequate operator skill. Potential complications such as flap necrosis, bleeding, or wound dehiscence may occur if proper surgical principles are not followed, although none were observed in the present series.

Within the limitations of this case series, it can be concluded that Z-plasty and V-Y plasty appear to be promising alternatives in selected clinical situations compared to conventional frenectomy, particularly in patients with high esthetic demands. An interdisciplinary approach combining periodontal surgery with orthodontic treatment remains essential for achieving optimal and stable outcomes in the management of midline diastema associated with aberrant frenum attachments.

## Conclusions

Within the limitations of this case series, frenectomy procedures using both conventional and advanced plastic surgical techniques effectively eliminated aberrant labial frenum attachments and facilitated subsequent orthodontic management. However, advanced techniques such as Z-plasty and V-Y plasty may provide improved esthetic and functional outcomes compared to the conventional approach, primarily due to tension-free closure, increased vestibular depth, and reduced scar formation. In contrast, conventional frenectomy, although simple and reliable, showed a greater tendency for scar formation and healing by secondary intention. Z-plasty and V-Y plasty, as periodontal plastic surgical procedures, may allow better tissue redistribution and improved healing, making them particularly useful in cases with high esthetic demand. Careful case selection, surgical expertise, and an interdisciplinary approach remain essential for achieving potentially favorable short-term outcomes in the management of midline diastema associated with aberrant frenum attachments.
